# Translation and Cultural Adaptation of the Measure Yourself Concerns and Wellbeing (MYCaW) Questionnaire Into German: Protocol for the iSWOP Study

**DOI:** 10.2196/74288

**Published:** 2025-10-23

**Authors:** Anja Thronicke, Lisa Schille, Katja Adie, Christian Junghanss, Shiao Li Oei, Sophia Johnson, Juliane Roos, Friedemann Schad

**Affiliations:** 1 Research Institute Havelhöhe, Hospital Gemeinschaftskrankenhaus Havelhöhe, Kladower Damm 221, Berlin, 14089, Germany, +49 30-36501 ext 5015; 2 Care of the Elderly Department, Royal Cornwall Hospital, Treliske, Truro, United Kingdom; 3 Department of Medicine, Clinic III - Hematology, Oncology, Palliative Medicine, Rostock University Medical Center, Rostock, Germany; 4 Interdisciplinary Oncology and Palliative Care, Hospital Gemeinschaftskrankenhaus Havelhöhe, Berlin, Germany

**Keywords:** health-related quality of life, HRQL, patient-reported outcome measures, PROM, cross-cultural adaptation, oncological patients, psychometric validation

## Abstract

**Background:**

The growing population of cancer survivors faces persistent physical and emotional challenges that significantly impact health-related quality of life (HRQL). To address these multifaceted needs, robust and culturally adapted patient-reported outcome measures (PROMs), such as the Measure Yourself Concerns and Wellbeing (MYCaW) questionnaire, are essential for understanding and improving survivors’ subjective experiences.

**Objective:**

This protocol aimed to outline the systematic translation and cultural adaptation of the MYCaW questionnaire into German. The MYCaW questionnaire, a PROM, is designed to capture individualized concerns and assess overall well-being, particularly in cancer care settings. By adhering to common guidelines, this research will provide a tool for assessing individualized concerns and patient needs among German-speaking patients with cancer.

**Methods:**

Following International Society for Pharmacoeconomics and Outcomes Research (ISPOR) guidelines, this study will use a structured methodology involving forward and backward translation, expert review, patient review process, and preliminary validation to ensure linguistic and cultural equivalence. This study is approved by the ethics committee of the Medical Association Berlin (reference Eth-27/10). Construct validity will be assessed through comparison with the European Organisation for Research and Treatment of Cancer—Quality of Life Questionnaire, Core 30 (EORTC QLQ-C30) and the MIDOS (Minimal Documentation System; in German: Minimales Dokumentationssystem) questionnaire, to evaluate both quality of life and symptom burden.

**Results:**

Funding of the study was obtained in January 2023. Patient recruitment started in the first quarter of 2023, and the cognitive debriefing phase is ongoing. Validation with the larger patient sample (N=120) is scheduled to conclude in the fourth quarter of 2025, with publication of the study results anticipated in the first quarter of 2026. The adaptation process will include translation, expert review, and cognitive debriefing with patients and health care professionals to ensure linguistic clarity and cultural relevance.

**Conclusions:**

The translation and adaptation of MYCaW into German will contribute to expanding the availability of validated PROMs for German-speaking populations. By following rigorous international guidelines, this study aims to produce a reliable, culturally appropriate, and linguistically adapted German version of the MYCaW questionnaire for assessing patient concerns and well-being in oncology and supportive care settings. Future validation studies will be necessary to assess the psychometric properties of the adapted questionnaire and its applicability in clinical and research contexts. Potential challenges, such as maintaining conceptual equivalence in translation and ensuring broad representativeness in the validation process, will be addressed through iterative refinement. Once validated, the German MYCaW will provide a valuable resource for patient-centered research and care, helping to capture individualized concerns that might be overlooked by standardized instruments.

## Introduction

A growing number of people are living with cancer or have survived it, making the well-being of these individuals an important area for research [1]. In addition to the advances in early detection and the increasing efficacy of oncology therapies, understanding and improving the health-related quality of life (HRQL) of patients with cancer has gained increasing attention. Many patients with cancer face persistent physical and emotional challenges long after completing primary therapy. Long-term and late effects of cancer treatments—such as chronic pain, cognitive dysfunction, and severe fatigue—can significantly impact HRQL [2,3]. These impairments often necessitate comprehensive strategies to address the multifaceted needs of oncology patients.

The assessment of HRQL relies on validated patient-reported outcome measures (PROMs) [4]. These tools provide invaluable insights into patients’ subjective experiences, aiding clinicians and researchers in identifying specific areas requiring intervention [5]. One such tool, the Measure Yourself Concerns and Wellbeing (MYCaW) questionnaire, is recognized for its ability to capture individualized concerns and overall well-being [6]. MYCaW uses a patient-centered approach, allowing participants to report their primary concerns and assess their well-being, making it particularly useful in integrative oncology settings. The MYCaW questionnaire was first introduced in 2006 by Paterson et al [6] at the University of Bristol with support from the Medical Research Council Health Services Research Collaboration. Earlier work on MYCaW at the Bristol Cancer Help Center was recognized for its impact, earning a place on the shortlist and winning the Healthcare and Medical Research category at the 2003 Charity Awards. In 2020, Polley et al [7] and Seers et al [8] took over responsibility for MYCaW and Measure Yourself Medical Outcome Profile (MYMOP) leading to the foundation of Meaningful Measures Ltd [7-9] to manage the licensing of the questionnaire [10], while providing tailored guidance on capturing person-centered outcomes.

Most PROMs focus on predefined domains such as symptom burden, functioning, or general quality of life [4,11,12]. While these instruments are essential, they often fail to capture the individualized concerns that many patients identify as most pressing. In integrative oncology, where care extends beyond symptom control to include psychosocial and existential dimensions, such patient-defined concerns are particularly relevant [13,14]. Studies have shown that individualized priorities—such as fear of recurrence, family-related worries, or coping with identity changes—are frequently underrepresented in standardized PROMs [15]. Internationally, the MYCaW questionnaire has proven effective in addressing this gap and has been successfully adapted into several languages [16-20]. However, no validated German version currently exists. This represents a significant unmet need, as German-speaking oncology patients and researchers lack access to a culturally adapted tool for systematically documenting patient priorities and for enabling comparability across international studies.

The cross-cultural adaptation and validation of PROMs are essential to ensure their relevance and applicability across different languages and cultural contexts [21]. Translation is a critical first step in this process, requiring not only linguistic precision but also cultural sensitivity to maintain the questionnaire’s conceptual equivalence and reliability. To guide this process, the widely recognized framework by Beaton et al [21] outlines a systematic multistep methodology involving forward and backward translations, expert review, and patient review processes to ensure conceptual equivalence between the original and adapted versions. In addition, the International Society for Pharmacoeconomics and Outcomes Research (ISPOR) principles for good practices in translation and cultural adaptation emphasize maintaining linguistic precision and cultural sensitivity [22], while the Consensus-based Standards for the selection of health status Measurement Instruments (COSMIN) guidelines provide recommendations for assessing the measurement properties of translated instruments [23], such as validity, reliability, and cross-cultural equivalence. Together, these frameworks ensure that adapted tools are both culturally relevant and psychometrically sound.

This study protocol outlines the process of translating the MYCaW questionnaire into German and aims to provide a detailed framework for its cultural adaptation. By achieving a validated German version, this research aims to facilitate its use among German-speaking patients with cancer, enabling a deeper understanding of their self-reported individualized concerns and needs.

The primary objective of this protocol is to describe the methodology used in the translation and adaptation of the MYCaW questionnaire into German. Ultimately, the study aims to contribute to the broader field of patient-centered oncology care by enabling more accurate and culturally appropriate assessments of HRQL in German-speaking settings.

## Methods

### Study Design

This study will follow the ISPOR guidelines for the translation and cultural adaptation of patient-reported outcome measures [22] and the COSMIN guidelines for assessing cross-cultural validity and measurement invariance [23]. The process includes preparation, translation, patient review, and validation. The study protocol was written using the SPIRIT (Standard Protocol Items: Recommendations for Interventional Trials) reporting guidelines [24].

### Study Setting

This study is conducted by the Research Institute Havelhöhe (Forschungsinstitut Havelhöhe) at the hospital Gemeinschaftskrankenhaus Havelhöhe in Berlin within the Network Oncology [25].

### Ethical Considerations

This study adheres to the principles of the Declaration of Helsinki and has received approval from the Ethics Committee of the Berlin Medical Association (Ethik-Kommission der Ärztekammer Berlin) under reference Eth-27/10. This trial is registered in the German Clinical Trials Register (DRKS00013335; registered on November 27, 2017). Written informed consent will be obtained from all participants before enrollment. Any significant protocol deviations or modifications will be reported to the Ethics Committee and the German Clinical Trials Register. A completed SPIRIT 2013 checklist is provided (see Checklist 1). Patients and the public were not involved in the design, reporting, or dissemination plans of the study protocol of this research but will be involved in the conduct of the outlined study in the future during the debriefing and validation process. All participants receive written and verbal information about the study, including its purpose, procedures, risks, and benefits, and will give written informed consent before participation. They may withdraw at any time without consequences. Participant privacy and confidentiality will be strictly protected. Identifiable information will be stored separately from research data on secure, password-protected institutional servers accessible only to authorized study personnel. Data will be pseudonymized for analysis, publication, and possible sharing and retained only as long as legally and scientifically required. Participation involves minimal risk and no invasive procedures. No financial incentives are offered. This protocol complies with JMIR’s ethical reporting guidance

### Translation Process

**Figure 1.**
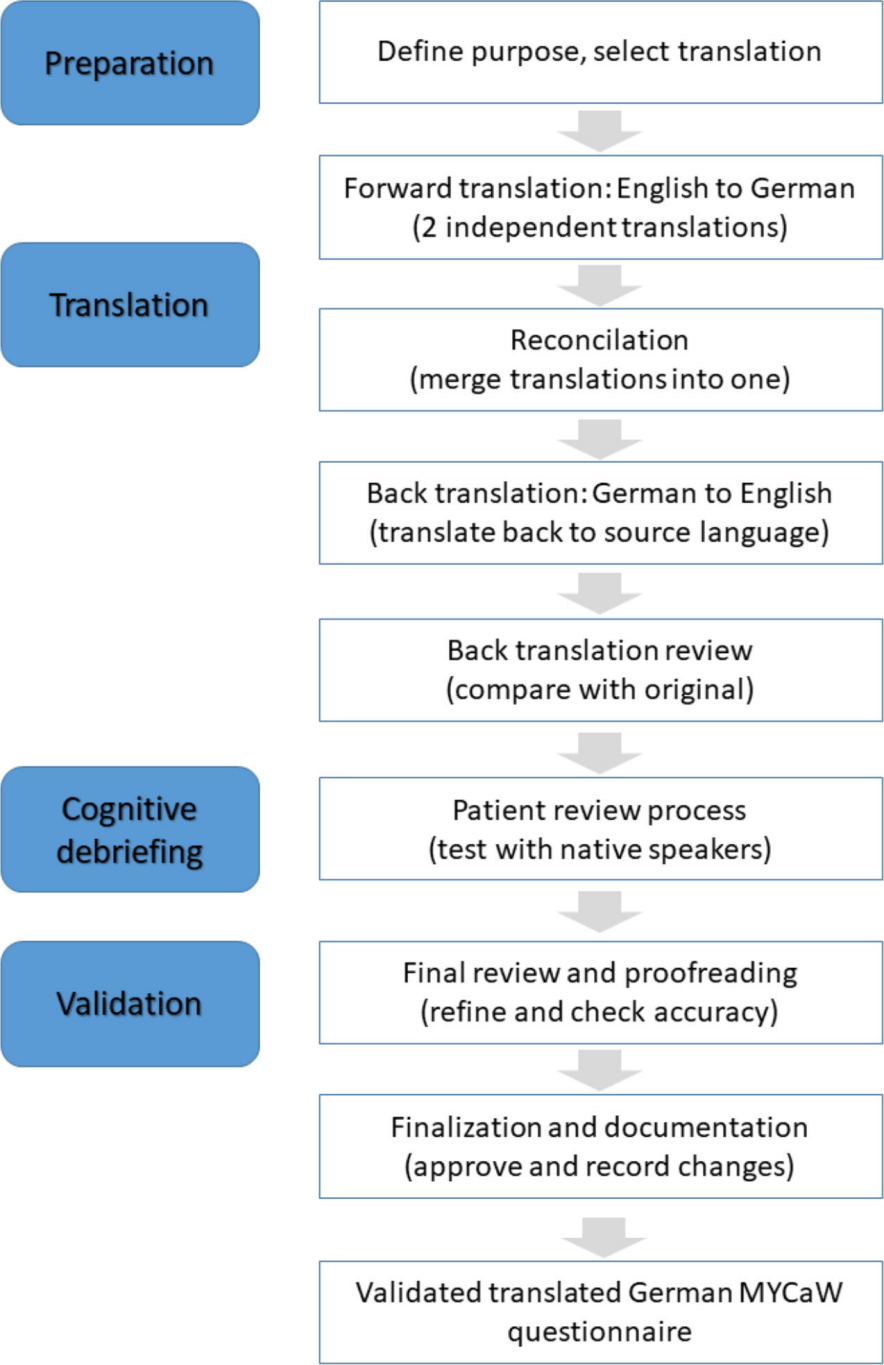
Flow chart of the translation process according to International Society for Pharmacoeconomics and Outcomes Research (ISPOR) guidelines. MYCaW: Measure Yourself Concerns and Wellbeing.

Figure 1 shows the flowchart illustrating the systematic process of the questionnaire translation into German language following the ISPOR guidelines. The steps ensure linguistic and conceptual equivalence between the original and translated versions, maintaining validity and reliability. The translation process involves 2 independent bilingual translators (native German speakers fluent in English) who will produce 2 German versions of the MYCaW questionnaire. These versions will be, after discussion of discrepancies, combined into a single German draft (see Figure 1).

The translators will focus on maintaining conceptual equivalence rather than literal translation, considering cultural nuances and medical terminology.

This process is followed by 2 back-translations into English with the help of 2 native independent English speakers fluent in German. The backward translations will be compared with the original MYCaW questionnaire to identify discrepancies (see Figure 1). A reconciliation meeting including translators and a third bilingual expert will be held to resolve semantic, idiomatic, and conceptual discrepancies, ensuring conceptual equivalence. A panel of experts, including oncologists, psychologists, and patient representatives, will review all versions to ensure linguistic and cultural accuracy. The panel will finalize a pretest version of the German MYCaW questionnaire.

### Participant Review

A total of 15 patients with cancer will be recruited for cognitive debriefing based on diversity in age, cancer type and stage, treatment history, and educational background. This purposive sampling approach is intended to enhance representativeness within the single-site setting and to capture a broad spectrum of patient perspectives. Inclusion criteria are providing written informed consent, being 18 years and/or older, having a confirmed (any) cancer diagnosis, and having sufficient German language proficiency. Exclusion criteria are no written informed consent and cognitive impairment of the patient. The pretest version will be evaluated by these patients for its clarity, cultural relevance, and ease of understanding. Semistructured interviews will be conducted to gather participants’ feedback on specific items (see Figure 1).

### Analysis of Cognitive Debriefing Interviews

Cognitive debriefing interviews will be audio-recorded, transcribed verbatim, and analyzed using qualitative content analysis according to Mayring [26]. In addition, 2 researchers will independently code the transcripts, combining deductive categories (eg, clarity, cultural relevance, and comprehensibility) with inductive categories emerging from the data. Discrepancies will be resolved by discussion or with a third reviewer. Findings will be summarized in matrices to identify key issues and inform refinement of the German MYCaW version.

### Validation Phase and Psychometric Testing

The subsequent validation phase will include approximately 120 participants from the same site, but purposive sampling will again be applied to ensure heterogeneity in demographic and clinical characteristics. This sample aligns with COSMIN recommendations for studies assessing psychometric properties. Specifically, it meets thresholds considered “very good” for evaluating internal consistency and construct validity and provides sufficient power for exploratory analyses of structural validity and reliability. Content validity will be assessed through patient feedback, which helps ensure that the questionnaire items are relevant, comprehensive, and representative of the real experiences of the patients with cancer. This will involve qualitative methods such as interviews or surveys to compare MYCaW content with participant-reported concerns and well-being issues. Internal consistency will be evaluated using Cronbach α, with a commonly accepted threshold for good internal consistency of 0.7 or higher [27]. This will be calculated for each subscale of the MYCaW questionnaire to ensure that items within each dimension of HRQL are consistent and reliable in measuring the same underlying construct. To ensure consistency and reliability in the qualitative analysis of patient concerns, all coders will undergo structured training in the use of the adapted MYCaW coding framework. This training will include familiarization with category definitions, practice sessions using sample data, and calibration exercises to align interpretation. Coding discrepancies between raters will be addressed through a consensus process involving joint review and discussion. If no consensus can be reached, a third independent expert will be consulted to resolve the disagreement.

To assess construct validity, the MYCaW questionnaire that will be compared with the EORTC-QLQ C30 is the European Organisation for Research and Treatment of Cancer—Quality of Life Questionnaire, Core 30 (EORTC-QLQ C30) [11], and the MIDOS questionnaire [28] for HRQL measures will be used to examine whether the MYCaW questionnaire captures similar constructs and whether changes in MYCaW scores align with changes in other established measures. This approach helps to ensure that the MYCaW questionnaire is measuring HRQL in a manner consistent with these validated tools. While the EORTC-QLQ C30 evaluates HRQL across multiple domains (symptom scales and functioning scales), the MIDOS assesses symptom burden in palliative or supportive care contexts. This dual comparison allows analysis of both standardized and patient-prioritized concerns.

### Contingency Planning

To mitigate potential recruitment delays, the study timeline allows for flexible enrollment extensions. Collaboration with clinical teams at the study site will be strengthened to ensure participant identification and recruitment. These steps are aimed at ensuring timely recruitment without compromising statistical validity.

### Timeline and Milestone Plan of the Study

**Figure 2.**
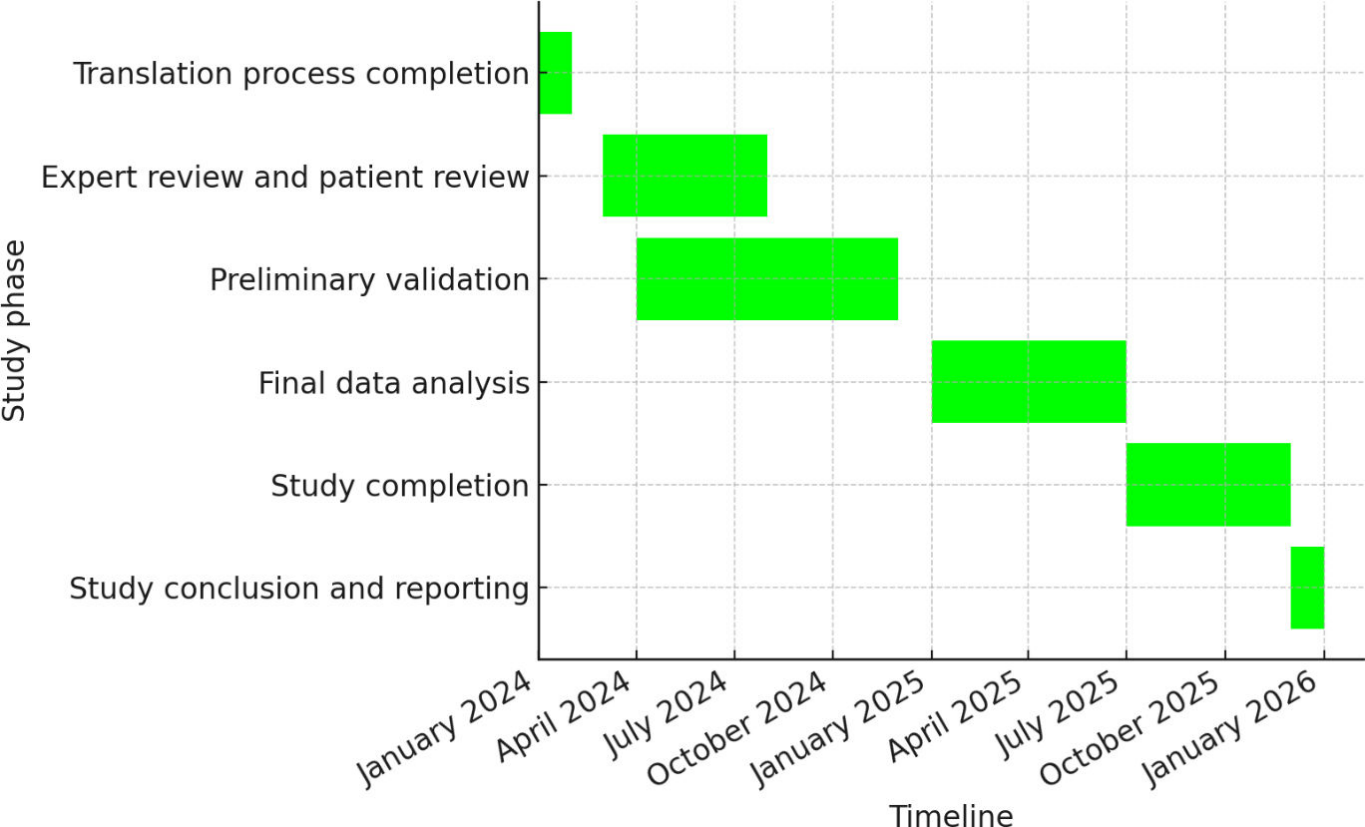
Milestone plan of the iSWOP (Implementierung eines Fragebogens zur Erfassung von Sorgen und Wohlbefinden bei onkologisch-palliativen PatientInnen) study to ensure adequate enrollment, Q3–Q4 2024 allows for potential extensions in the event of recruitment challenges.

Table 1 presents the timeline of the MYCaW translation and validation process. Key milestones and projected completion dates are highlighted, with further details available in Figure 2 (Gantt chart). The finalized German version will undergo preliminary testing with a larger sample to assess reliability and validity (see Table 1). The psychometric properties to be evaluated include content validity, internal consistency, and construct validity. These psychometric evaluations are critical to ensuring that the translated MYCaW questionnaire is both reliable and valid for assessing HRQL among German-speaking oncology patients.

Figure 2 presents the Gantt chart outlining the timeline of the MYCaW translation process. Key milestones and projected completion dates are highlighted, with further details available in Table 1.

**Table 1.** Timeline of the study.

Phase and timeline	Translation process
Year 2023: Translation process completion (Phase 1)	
Quarter 4, 2023 to Quarter 2, 2024	Finalize the German MYCaW^a^ version after translation, reconciliation, and expert review.
Year 2024: Patient review process and preliminary validation (Phase 2‐3)	
Quarter 2, 2024 to Quarter 3, 2024	Patient review process. Test the prefinal version with 15 patients, conduct interviews, and refine based on feedback.
Quarter 3, 2024 to Quarter 4, 2024^b^	Finalize the German version, begin validation with a larger sample (N=120) of participants, and assess psychometric properties (content validity, internal consistency, and construct validity).
Year 2025: Data analysis, final testing, and study conclusion (Phase 4‐6)	
Quarter 4, 2024 to Quarter 3, 2025	Continue validation and finalize data collection. Complete recruitment, conduct final psychometric analysis, and review preliminary results.
Quarter 3, 2025 to Quarter 4, 2025	Conclude study and finalize participant follow-up. Publish findings, present results, and complete study.
December 2025: Study officially ends.	

a MYCaW: Measure Yourself Concerns and Wellbeing.

b Contingency planning allows for time extensions in quarter 3–quarter 4, 2024, to allow for potential recruitment delays.

## Results

The study was funded in January 2023. Recruitment began in the first quarter of 2023, with cognitive debriefing currently being underway. Validation with a larger patient sample (N=120) is projected to be completed by the fourth quarter of 2025, with results expected for publication in the first quarter of 2026. A standardized coding framework will be developed for analyzing patient concerns, with interrater reliability assessed to ensure consistency. The final German MYCaW version is expected to maintain the conceptual integrity of the original while being accessible and meaningful for German-speaking oncology patients.

## Discussion

### Principal Findings

This study outlines a systematic approach to the translation and cultural adaptation of the MYCaW questionnaire into German, which is essential for capturing individualized patient concerns in integrative oncology. MYCaW has been widely used in various research settings to assess patient-reported concerns and overall well-being. Its flexible and patient-centered design allows individuals to express their most pressing issues, making it particularly suitable for integrative oncology and supportive care [6]. Across health care contexts, MYCaW has shown value in complementing standardized HRQL tools.

Several studies have used MYCaW to explore the impact of interventions, assess patient priorities, and track changes in well-being over time [6,20,29,30]. Previous research demonstrated that MYCaW captures nuanced, patient-specific concerns that standardized questionnaires may overlook, thereby facilitating tailored interventions that may improve overall quality of life [6,8]. For example, in a palliative care setting, MYCaW’s open-ended design provided valuable qualitative insights into patient needs, and health care professionals reported it as easy to use and beneficial for patient-focused discussions [29]. A standardized coding framework with 5 super-categories and 36 specific categories was developed to analyze concerns, achieving high interrater reliability (κ=0.905) [29]. In addition, studies comparing MYCaW with established HRQL measures, such as the EORTC QLQ-C30, also found it complements existing tools by offering a more individualized assessment [30].

Like MYCaW, the precursor questionnaire MYMOP has also been used extensively to evaluate patient-centered outcomes. While MYMOP focuses on symptom-specific concerns and treatment effects, both instruments share a common philosophy of prioritizing patient voice and individualized health concerns [31]. MYCaW is particularly relevant in psycho-oncology due to its focus on well-being.

Regarding translation and cultural adaptation, MYCaW has already been successfully adapted into several languages, including Danish, Polish, Hebrew, Romanian, Somali, Spanish, and Turkish [16,17]—following rigorous methodologies. These efforts underscore the importance of careful linguistic validation to preserve validity and reliability [32]. The German translation and adaptation of MYCaW presented here follows internationally recognized frameworks (ISPOR and COSMIN) [22] and the Meaningful Measures protocol, ensuring conceptual equivalence and cultural relevance [21]. Expert review, reconciliation meetings, and cognitive debriefing interviews with patients are designed to secure both linguistic fidelity and relevance to the target population. Validation against 2 widely recognized PROMs, the EORTC QLQ-C30 and the MIDOS questionnaire, will allow for a nuanced analysis of MYCaW’s psychometric performance and its alignment with both functional quality-of-life and symptom-specific metrics. This dual approach provides a robust framework for assessing construct validity in the German oncology context. The German MYCaW adaptation aligns with similar initiatives in other languages, which have emphasized the need for iterative refinement through patient and expert feedback [33]. These processes are essential for ensuring that the instrument effectively captures the lived experiences of diverse patient populations.

The study has several strengths, notably its systematic methodology and adherence to international guidelines. By incorporating cognitive debriefing and semistructured interviews, the protocol ensures that the German MYCaW will be linguistically accurate and culturally relevant to German-speaking patients with cancer. Once validated, it will provide health care professionals with a valuable tool for addressing individualized concerns and needs in oncological care. Nevertheless, some limitations remain. The cognitive debriefing phase includes 15 participants, sufficient to identify major issues but not necessarily capturing all nuances in a diverse population. Although purposive sampling will be applied to maximize heterogeneity, recruitment is restricted to a single site, which limits representativeness across the German-speaking cancer population. While the German version may also be linguistically appropriate for Austria and Switzerland, cultural relevance in these settings has not been tested and will require future studies. Patient and public involvement in this protocol has been limited, though their perspective will be integrated during debriefing and validation; in the future, earlier engagement in study design could enhance cultural sensitivity. Finally, inherent biases in forward-backward translation may influence conceptual equivalence, despite the safeguards of expert panel and reconciliation meetings. Future studies should focus on larger-scale validation and psychometric testing to further refine the German MYCaW and enhance its applicability in routine clinical practice [34].

Several risks to validity must also be acknowledged. A small debriefing sample may overlook issues of comprehension or cultural resonance; this will be mitigated through purposive sampling to ensure variation in age, diagnosis, and educational background. Single-site recruitment limits generalizability, but careful documentation of participant characteristics will support assessment of transferability. The qualitative analysis of interviews is inherently interpretative; to strengthen credibility, 2 independent coders will apply the Mayring qualitative content analysis, with discrepancies resolved through discussion or arbitration by a third reviewer. Conceptual equivalence in translation is another challenge; this will be minimized through systematic reconciliation, expert panel review, and iterative patient feedback.

### Conclusion

In conclusion, MYCaW has been shown internationally to be a valuable tool for assessing individualized patient concerns and well-being. Its successful application in diverse settings highlights its relevance, and the ongoing translation and adaptation efforts aim to expand accessibility across different linguistic and cultural contexts. The German translation and adaptation will address an important gap by providing a culturally appropriate instrument for patient-centered oncology research and care, thereby contributing to a more comprehensive understanding of cancer survivors’ needs. While the protocol is methodologically robust, larger multicenter studies will be needed to confirm generalizability across the wider German-speaking population. Future projects should also involve patients and the public more actively at earlier stages of study design. Finally, the methodology described here may serve as a blueprint for similar cross-cultural adaptation studies.

### Trial Status and Protocol Amendments

The current protocol version is 1.0, dated from January 17, 2023. The trial is ongoing and currently enrolling. The first participant was enrolled on January 23, 2023, and recruitment is expected to be completed in the fourth quarter of 2025 with the follow-up completed by the end of 2025. Any amendments to the study protocol (eg, changes in recruitment procedures, sample size, or analysis plan) will be documented in a version-controlled protocol file, reported to the ethics committee, and prospectively updated in the trial registry (DRKS00013335). Major changes will also be transparently reported in subsequent publications.

### Dissemination

The findings will be presented at international and national scientific conferences such as the European Society for Medical Oncology congress, the European Congress of Integrative Medicine, the German Cancer Congress of the Cancer Society (Deutscher Krebskongress) or the congress of the German Society of Oncology and Hematology. For scientific publication, the paper will be submitted to peer-reviewed journals with a focus on oncology, integrative oncology, or psycho-oncology, such as *BMC Cancer*, *Supportive Care in Cancer*, or *The Oncologist*. Targeted communication with stakeholders in the patient community involves presentations at patient-oriented congresses such as the Cancer Patients Europe Annual Conference [35] or the European Association for Cancer Research Congress Patient Involvement in Cancer Research [36]. Furthermore, results will be communicated through lay summaries published in newsletters of the hospital or of patient organizations, for example, Haus der Krebs-Selbsthilfe—Bundesverband eV, Leben nach Krebs eV [37], and Deutsche Stiftung für Junge Erwachsene mit Krebs [38], and disseminated digitally via the hospitals' social media channel as well as these organizations. These efforts will ensure that findings are accessible and understandable for patients, caregivers, and the wider community.

## Supplementary material

10.2196/74288 Checklist 1 SPIRIT 2025 checklist.

## Abbreviations

COSMIN: Consensus-based Standards for the selection of health status Measurement Instruments
EORTC-QLQ C30: European Organisation for Research and Treatment of Cancer—Quality of Life Questionnaire, Core 30
HRQL: health-related quality of life
ISPOR: International Society for Pharmacoeconomics and Outcomes Research
MYCaW: Measure Yourself Concerns and Wellbeing
MYMOP: Measure Yourself Medical Outcome Profile
PROM: patient-reported outcome measure
SPIRIT: Standard Protocol Items: Recommendations for Interventional Trials
